# Social determinants of health and disease in companion dogs: a cohort study from the Dog Aging Project

**DOI:** 10.1093/emph/eoad011

**Published:** 2023-05-13

**Authors:** Brianah M McCoy, Layla Brassington, Kelly Jin, Greer A Dolby, Sandi Shrager, Devin Collins, Matthew Dunbar, Dog Aging Project Consortium, Joshua M Akey, Brooke Benton, Elhanan Borenstein, Marta G Castelhano, Amanda E Coleman, Kate E Creevy, Kyle Crowder, Matthew D Dunbar, Virginia R Fajt, Annette L Fitzpatrick, Unity Jeffery, Erica C Jonlin, Matt Kaeberlein, Elinor K Karlsson, Kathleen F Kerr, Jonathan M Levine, Jing Ma, Robyn L McClelland, Daniel E L Promislow, Audrey Ruple, Stephen M Schwartz, Sandi Shrager, Noah Snyder-Mackler, Katherine Tolbert, Silvan R Urfer, Benjamin S Wilfond, Audrey Ruple, Noah Snyder-Mackler

**Affiliations:** School of Life Sciences, Arizona State University, Tempe, AZ, USA; Center for Evolution and Medicine, Arizona State University, Tempe, AZ, USA; School of Life Sciences, Arizona State University, Tempe, AZ, USA; Center for Evolution and Medicine, Arizona State University, Tempe, AZ, USA; Allen Institute for Brain Science, Seattle, WA, USA; Department of Biology, University of Alabama at Birmingham, Birmingham, ALUSA; Collaborative Health Studies Coordinating Center, Department of Biostatistics, University of Washington, Seattle, WA, USA; Department of Sociology, University of Washington, Seattle, WA, USA; Center for Studies in Demography & Ecology, University of Washington, Seattle, WA, USA; Lewis-Sigler Institute for Integrative Genomics, Princeton University, Princeton, NJ, USA; Department of Laboratory Medicine and Pathology, University of Washington School of Medicine, Seattle, WA, USA; Department of Clinical Microbiology and Immunology, Sackler Faculty of Medicine, Tel Aviv University, Tel Aviv, Israel; Cornell Veterinary Biobank, College of Veterinary Medicine, Cornell University, Ithaca, NY, USA; Department of Small Animal Medicine and Surgery, College of Veterinary Medicine, University of Georgia, Athens, GA, USA; Department of Small Animal Clinical Sciences, Texas A&M University College of Veterinary Medicine & Biomedical Sciences, College Station, TX, USA; Department of Sociology, University of Washington, Seattle, WA, USA; Center for Studies in Demography and Ecology, University of Washington, Seattle, WA, USA; Department of Veterinary Physiology and Pharmacology, Texas A&M University College of Veterinary Medicine & Biomedical Sciences, College Station, TX, USA; Department of Family Medicine, University of Washington, Seattle, WA, USA; Department of Veterinary Pathobiology, Texas A&M University College of Veterinary Medicine & Biomedical Sciences, College Station, TX, USA; Department of Laboratory Medicine and Pathology, University of Washington School of Medicine, Seattle, WA, USA; Department of Laboratory Medicine and Pathology, University of Washington School of Medicine, Seattle, WA, USA; Bioinformatics and Integrative Biology, University of Massachusetts Chan Medical School, Worcester, MA, USA; Department of Biostatistics, University of Washington, Seattle, WA, USA; Department of Small Animal Clinical Sciences, Texas A&M University College of Veterinary Medicine & Biomedical Sciences, College Station, TX, USA; Division of Public Health Sciences, Fred Hutchinson Cancer Research Center, Seattle, WA, USA; Department of Biostatistics, University of Washington, Seattle, WA, USA; Department of Laboratory Medicine and Pathology, University of Washington School of Medicine, Seattle, WA, USA; Department of Population Health Sciences, Virginia-Maryland College of Veterinary Medicine, Virginia Tech, Blacksburg, VA, USA; Epidemiology Program, Fred Hutchinson Cancer Research Center, Seattle, WA, USA; Collaborative Health Studies Coordinating Center, Department of Biostatistics, University of Washington, Seattle, WA, USA; School of Life Sciences, Arizona State University, Tempe, AZ, USA; Department of Small Animal Clinical Sciences, Texas A&M University College of Veterinary Medicine & Biomedical Sciences, College Station, TX, USA; Department of Laboratory Medicine and Pathology, University of Washington School of Medicine, Seattle, WA, USA; Treuman Katz Center for Pediatric Bioethics, Seattle Children’s Research Institute, Seattle, WA, USA; Department of Population Health Sciences, Virginia-Maryland College of Veterinary Medicine, Virginia Tech, Blacksburg, VA, USA; School of Life Sciences, Arizona State University, Tempe, AZ, USA; Center for Evolution and Medicine, Arizona State University, Tempe, AZ, USA; School for Human Evolution and Social Change, Arizona State University, Tempe, AZ, USA

**Keywords:** social determinants, health and disease, companion dogs, aging

## Abstract

Exposure to social environmental adversity is associated with health and survival across many social species, including humans. However, little is known about how these health and mortality effects vary across the lifespan and may be differentially impacted by various components of the environment. Here, we leveraged a relatively new and powerful model for human aging, the companion dog, to investigate which components of the social environment are associated with dog health and how these associations vary across the lifespan. We drew on comprehensive survey data collected on 21,410 dogs from the Dog Aging Project and identified five factors that together explained 33.7% of the variation in a dog’s social environment. Factors capturing financial and household adversity were associated with poorer health and lower physical mobility in companion dogs, while factors that captured social support, such as living with other dogs, were associated with better health when controlling for dog age and weight. Notably, the effects of each environmental component were not equal: the effect of social support was 5× stronger than financial factors. The strength of these associations depended on the age of the dog, including a stronger relationship between the owner’s age and the dog’s health in younger as compared to older dogs. Taken together, these findings suggest the importance of income, stability and owner’s age on owner-reported health outcomes in companion dogs and point to potential behavioral and/or environmental modifiers that can be used to promote healthy aging across species.

## INTRODUCTION

The social environment is one of the strongest predictors of health and mortality in many mammals, where exposure to less social adversity is associated with a lower risk of disease and death [1]. In humans, higher socioeconomic status (SES) can lead to access to more resources, including healthcare, and a more predictable, less stressful environment that is associated with lower morbidity and mortality [2, 3]. Additionally, social isolation—another component of the social environment—has a stronger association with mortality than heavy smoking, heavy drinking and lack of exercise, when controlling for SES [3, 4]. These sociality-health links appear to be deeply rooted in evolutionary history: having been identified in species ranging from mice to primates [5–7].

How social adversity impacts older individuals is less clear and may vary based on timing, severity and perceived impact of exposure to adversity [8]. Environmental stability, which is composed of positive socioeconomic, built and natural environmental factors—such as higher household income, more social connections and access to greenspace, patterns overall health and disease risk in both early and late life [9]. In humans, urban environments with high residence turnover, high pollution levels and low economic status are associated with reduced physical mobility, increased disease risk and poor health outcomes [10] that can also worsen with age [11]. However, researchers are only beginning to explore how health and mortality associations with environmental factors vary across the lifespan in humans [12, 13]—knowledge that is necessary to optimize the distribution of resources and interventions to those who would benefit most. This gap in our knowledge is largely due to the long lives of humans and confounding factors that may be linked to other environmental factors (e.g. smoking).

To address this gap, we can turn to animal systems with similar biology, recent co-evolutionary history and environments to humans, but compressed into a much shorter lifespan. Companion dogs (*Canis familiaris*) offer a unique opportunity to look across the lifespan at how and when aspects of the social and physical environment may alter aging, health and survival. Dogs have been shown to be a powerful comparative model due to our shared biology, shared environment and significantly shorter lifespan [14–18]. Similar to humans, the major causes of death and disease in older dogs are cancer, cardiovascular incidences and immune-mediated diseases—making them a particularly important model for understanding the biology of aging in mammals [18–20]. Dogs are also genetically and phenotypically diverse due to hundreds of years of selective breeding [21, 22], and this genetic history and architecture have also made them a powerful model for investigating the genetic factors underlying complex traits, including many human diseases—many of which are associated with aging [23, 24].

Yet, despite our shared environments and passion for our companion dogs, we still know relatively little about the health impacts of the social environmental factors on companion animals, such as dogs. This gap in our knowledge is in large part due to a dearth of detailed social-environmental data available for large samples of dogs of all ages. We, therefore, have a limited understanding of how lived experiences might affect health and aging in dogs, which could: (1) inform how environmental factors impact human health and aging across the lifespan, and (2) help our companion animals live longer, healthier lives.

In humans, older individuals are often more vulnerable to adverse risk exposures due to a decreased ability to recover from exposures (‘resilience’), and a limited capacity for withstanding these exposures (‘reserve’) [25, 26]. For example, older individuals who experience a stressor such as infection or surgery are at increased risk of adverse outcomes later in life [25]. This association extends to companion dogs, who have been shown to have poorer outcomes for relatively common conditions at older ages as compared to younger ages even when medical and surgical treatments are provided [27, 28]. Additionally, in humans, social adversity such as social isolation and undernourishment early in life can have far-reaching effects on development and, subsequently, on adult health, reproduction and survival [29–33]. It is likely that social adversity such as reduced social companionship, less time spent outdoors and other exposures to environmental risk factors identified in human populations can impact adult dog health and aging as well. In fact, it has been shown that early-life malnutrition in dog populations can lead to long-term behavioral effects, like showing increased levels of fear and aggression, in addition to affected physical health in adulthood [34]. Thus, broader research into the impacts of social adversity on health and aging in companion animals is warranted.

Here, we examined factors related to the social environment of companion dogs to determine if there were associations with health and disease outcomes, as well as a measure of frailty (mobility), using data collected by the Dog Aging Project (DAP). We focused on these outcome measures because they all exhibit age-related changes (declines in health and mobility and increases in disease occurrences) in humans and other animals. In particular, mobility is a key phenotypic signature of frailty—the age-related increase of vulnerability to adverse outcomes. Mobility is associated with overall health [35, 36] and declines with age [37, 38] in both human and dog populations, suggesting that it can be used as a potential biomarker for aging and age-related disease risk. We hypothesized that exposure to higher levels of social environmental adversity in early adulthood would predict poorer age-associated health outcomes in this population of dogs, and that the strength of this effect would vary across the lifespan. Specifically, we predicted that dogs living in environments associated with adversity (i.e. low social integration, less stable environments and lower SES) would be less mobile, in poorer health and have more disease than more socially advantaged dogs within the population.

## METHODS

### Study population and recruitment

The Dog Aging Project (DAP) is a community science project that aims to understand how genes, lifestyle and the environment influence aging and disease outcomes [39, 40]. Study participants are recruited to DAP through word-of-mouth, mainstream media and social media. For this study, we drew on the first year of survey data released on 10 May 2021, from 27,541 dogs completed by owners on or before 31 December 2020. Study data were collected and managed using REDCap (Research Electronic Data Capture) electronic data capture tools hosted through the DAP [41, 42].

### Dog Aging Project survey data

The Health and Life Experiences Survey (HLES) includes a number of small surveys that collect information about dog demographic characteristics, physical activity, environment, dog behavior, diet, medications and preventatives, health status and owner demographic characteristics. The DAP Environment Dataset contains geographically defined data from secondary sources pertaining to the dog’s external environment. Environmental data are based on respondents’ primary and (where applicable) secondary address information, provided in the HLES owner contact form. Environmental metadata was generated from the US Census Bureau (2019) and the 2015–2019 American Community Survey 5-year Public Use Microdata Samples. Respondent addresses are geocoded and linked to existing data that characterize various aspects of the dog’s external environment. A total of 56,285 Environment data records (two per dog) are included in the 2020 Curated Data Release, reflecting all study participants who became DAP pack members on or before 31 December 2020, and released on 10 May 2021 (*n* = 27,541). We selected only the data corresponding to the primary residence information for the year 2020. We removed participants under 2 years of age in order to only include dogs that are fully grown, following the American Animal Hospital Association life stages guidelines [43]. After applying all these criteria, 21,410 dogs remained for downstream analysis. We excluded variables that were less than 98% complete and redundant variables that captured similar metrics (e.g. swimming/aerobic activity which was captured by overall activity level). Variables that were excluded due to missingness either lacked census data or the question did not apply to the owner (i.e. potential follow-up questions associated with a specific type of home heating method).

### Mobility

We generated a composite metric that could roughly capture age-related decline without someone carrying out an in-person physical examination of the dog. This metric was calculated by taking the z-score for each activity-related variable in the HLES (activity level, average activity intensity, on-leash walk frequency, aerobic activity frequency, physical games frequency, average daily activity and on leash walk average) then assigning each dog a score that corresponded to the average of their z-score on the activity-related variables.

### Disease instances

The disease instance outcome metric was generated by summing the number of owner-reported diseases for each dog. We excluded diseases that did not have a severe effect on overall dog health as described in [44] and detailed in SI Table 5.

### Stability

The stability metric is a combined variable that encompasses the percentage of the population in a census tract that was in the same home 1 year ago, the percent of homes that are owned by the occupant, and the percentage of the population born in the USA. Each dog received a stability score calculated from taking the z-score of these variables and averaging them. These data were collected from the US Census Bureau (2019). Some of the variables included in this metric are human-centric measures that will also capture the stability of a dog’s local environment.

### Factor analysis

For this study, we focused our analysis on the responses to 33 survey questions in the HLES. These 33 questions were selected a priori based on their link to at least one of the axes of the social determinants of health (e.g. financial, environmental, social connection; see SI Table 2 and methods). We then reduced the 33 survey questions into a smaller set of latent variables using exploratory factor analysis and following well-established guidelines [45]. Briefly, we first performed parallel analysis to determine the number of factors to retain then conducted factor analysis using the Psych package (version 2.0.8) in R [46]. After determining the optimal number of factors to retain, we then performed exploratory factor analysis using the default settings for the fa() function.

### Modeling of health-related variables

We modeled each of our health metrics (owner-reported health, disease instances and mobility score) as a function of all environmental factors, age and weight, as well as the interaction between age and each factor (Supplementary Fig. 4). We included weight in the model to control for variance in the health-related variables associated with breed ancestry, due to large inter-breed differences in dog size [20, 47]. We do not have genotypic data from the mixed-breed dogs, so could not control for breed ancestry directly. Due to their distributions and variable types, each health metric required a different linear modeling framework. We used an ordinal regression to model owner-reported health, which was scored on a Likert scale from 1 to 6. We modeled the count-based owner-reported disease instances using a generalized linear model with a Poisson link function. Mobility was modeled using a standard linear model. For all outcomes, we conducted sensitivity analyses that included modeling a dataset that only included mixed-breed dogs which was modeled in a mixed-effects framework that included breed as a random effect (SI Table 4, Supplementary Fig. 4).

### SEM confirmatory factor analysis

Confirmatory factor analysis was performed on a manually curated set of variables (described below) for use in the structural equation modeling analysis. This analysis was used to verify the structure of our observed set of variables. This was performed using the cfa() function under the Lavaan package (version 0.6-9) in R [48].

### Model specification

The latent factor structure was defined based on known relationships in the literature studied primarily in humans ([49, 50], SI Table 6). To specify the model for this study, we chose measured variables based on the following criteria, they: (1) had clear individual interpretability, (2) were likely to capture different aspects of the latent factor, (3) were continuously distributed, (4) did not have missing data and (5) together maximized sample variance as recorded in factor analysis. Our latent factor of finance was informed using measures from the census median income, percent living below the poverty line and percent of households making less than $100,000 US per year as well as owner-reported annual income range. The latent factor of the local environment was informed by census-tract population density, neighborhood walk score, percent of individuals in the same household 1 year ago, and percent of the population that was born in the USA. These variables were selected as measures that capture unique information about the dog’s neighborhood environment. Lastly, our social environment latent variable was informed by the measurable variables of routine hours per day with dogs, children and adults. We defined the structural equation model (SEM) using the Lavaan (version 0.6-9) package in R [48]; structural equation meta-models and model syntax were adapted from guidelines for a graph-theoretic implementation of structural equation modeling [51]. Prior to the implementation of the SEM, thirteen variables were standardized to a mean of zero and standard deviation of 1 to account for scale differences among variables, and models were run using unweighted least squares. Two models were tested, with the difference being the addition of a pathway in model two from finance to the environment. Fit for each of the two models was assessed through several metrics and associated cutoffs for ‘good’ agreement between data and model: root mean squared error of approximation (RMSEA, <0.08), comparative fit index (CFI, >0.9), standardized root-mean-squared residual (SRMR, <0.08) and the Tucker–Lewis index (TLI, >0.9) [52]. Comparative model fit was assessed using the corrected Akaike information criterion (AICc). Confirmatory factor analysis on both latent and indicator variables was conducted using the cfa function in R. All structural model visualizations were generated using the TidySEM and SEMplot packages in R. We generated two models; the first model (M1) describes direct paths from the latent factor of finance, environment and social interactions to our outcome of health (Supplementary Fig. 2). The second model (M2) describes all direct paths indicated in M1 but with the added indirect path between finance and environment (Supplementary Fig. 3). These two models were both hypothesized to reliably describe the system and were tested for model fit before interpreting the structural equation modeling analysis.

## RESULTS

### Owner survey data from a large cohort of companion dogs

**Figure 1.**
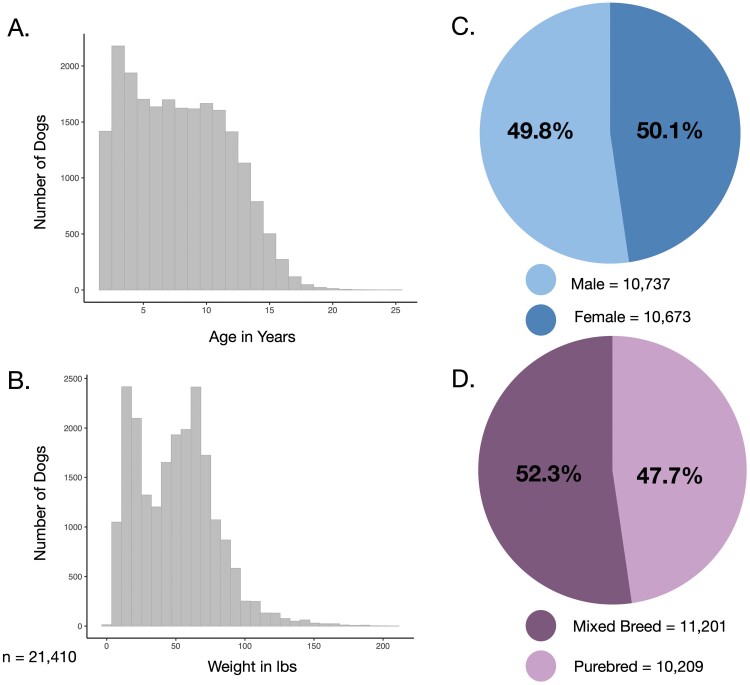
Demographic characteristics of dogs included in this study. Distribution of (a) age and (b) weight of dogs included in this study. The sample was roughly balanced by (c) sex and (d) mixed vs. purebred ancestry. Data are from the Dog Aging Project Health and Life Experience (HLES) Survey, 2019–2020

Our dataset included survey and health data from 21,410 dogs between the ages of 2 and 25.5 years old (Fig. 1a), that weighed between 2 and 210 lbs (Fig. 1b). We had a similar proportion of male (50.1%) and female dogs (49.8%; Fig. 1c), as well as purebred (52.3%) and mixed-breed dogs (47.7%; Fig. 1d). Given the low percentage of intact male and female dogs (4% and 2%, respectively), we chose to include them in our analyses. Additional demographic information on the dogs and their owners can be found in Supplementary information (SI Table 1, Supplementary Fig. 1).

### Social, financial and demographic factors

**Figure 2.**
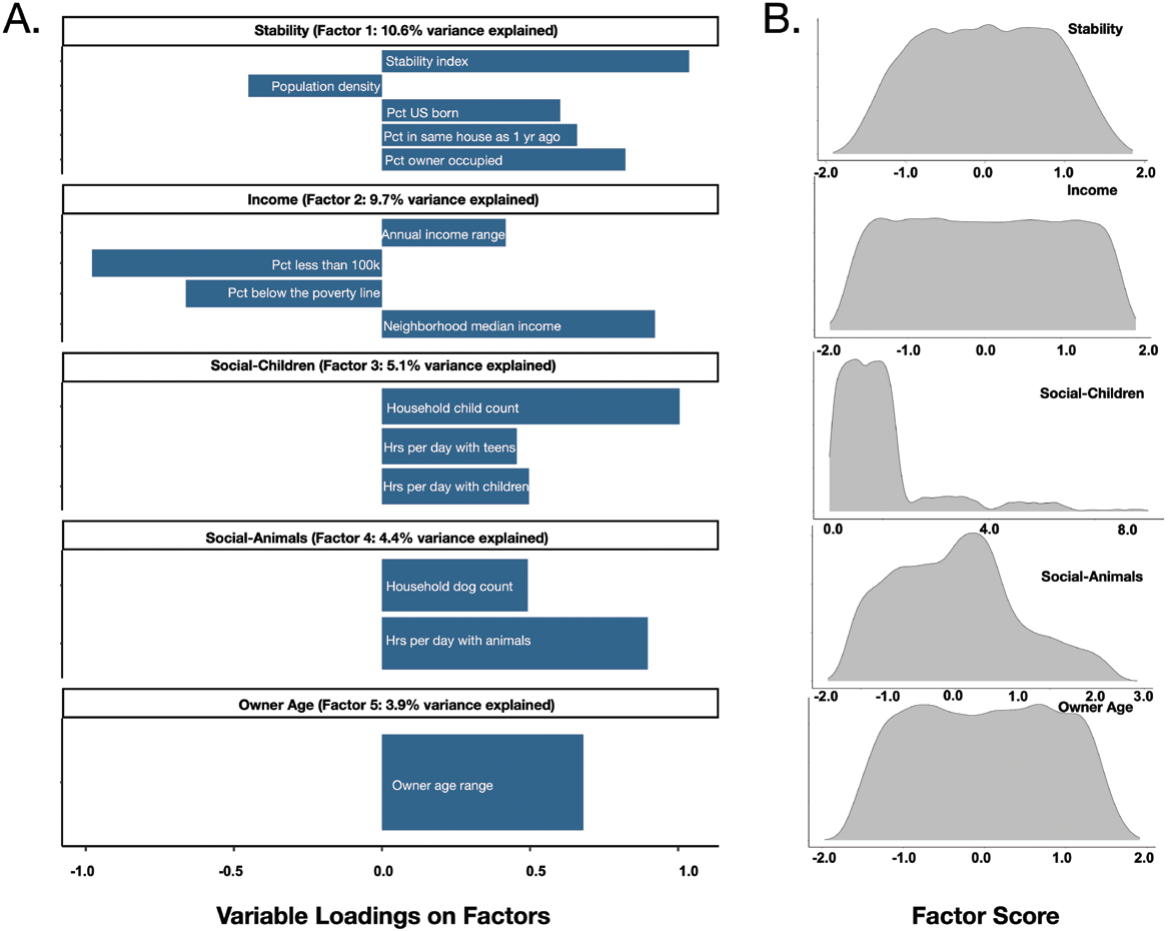
Five factors capture much of the social-environmental variation in the Dog Aging Project cohort. (a) Exploratory factor analysis revealed five factors that together explained 33.7% of the variance in survey responses. We named each factor based on the survey questions that loaded the strongest onto each factor (loading > |0.4|). (b) Distribution of factor scores for individual dogs for each of the five factors in our sample

We identified five factors, based on parallel analysis (see Methods), that collectively explained 33.7% of the variance in responses to the 33 survey questions, and then labeled the factors based on the survey questions that primarily contributed to each factor (loading >|0.4|): neighborhood stability (factor 1: 10.6% variance explained), total household income (factor 2: 9.7% variance explained), social-children (factor 3: 5.1% variance explained), social-animals (factor 4: 4.4% variance explained) and owner age (factor 5: 3.9% variance explained; Fig. 2).

### Household environment is associated with dog health, disease and mobility

#### Owner-reported dog health.

**Figure 3.**
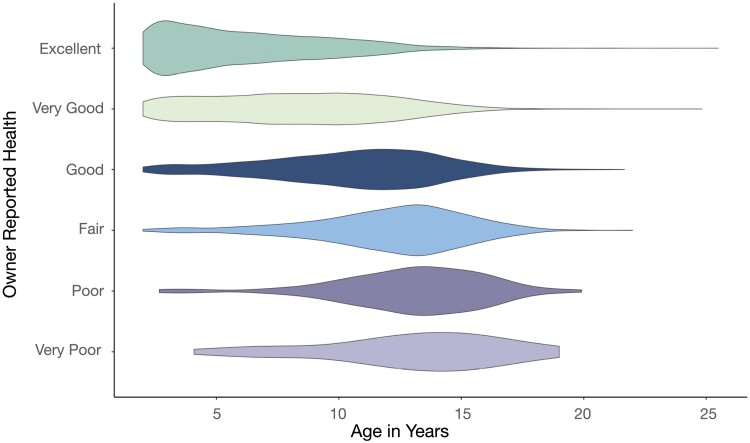
Owner-reported health is worse in older dogs. (a) Dog age was significantly associated with owner-reported health, such that older dogs were reported to be in poorer health compared to younger dogs controlling for dog weight (β_age_ = −0.421, *P* ≤ 2 × 10^−16^)

Owner-reported dog health was reported as an integer on a scale from 1 (‘very poor health’) to 6 (‘excellent health’). Since this is an unrefined measure of health, we first assessed its internal validity to capture dog health. As expected based on previous literature, older and heavier dogs were rated by their owners as being in the poorest health (β_age_ = −1.01, *P* ≤ 2 × 10^−16^; β_weight_ = −0.13, *P* = 6.45 × 10^−23^; Fig. 3 [53, 54]).

**Figure 4.**
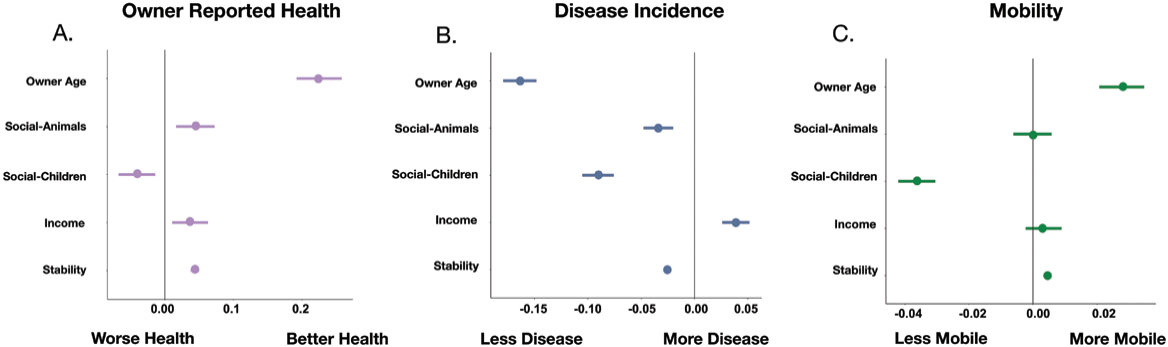
Social environment factors are associated with health, disease and mobility in companion dogs. Effect sizes of each environmental factor on (a) owner-reported dog health (ordinal model), (b) disease prevalence (cumulative number of diseases reported) (generalized linear model with Poisson link function) and (c) mobility (composite measure of activity level) (linear model). Points depict the effect sizes (β) of each factor and lines represent the 95% confidence intervals

We found that all five environmental factors were significantly associated with health outcomes (controlling for dog age and weight). Higher income was associated with better health (β_income_ = 0.037, *P* = 5.99 × 10^−3^), indicating that owners of higher SES rated their dogs as being healthier. Dogs with other dogs and/or other pets in their house were also rated as significantly healthier than dogs with fewer household companions (β_social-animals_ = 0.044, *P* = 1.20 × 10^−3^; Fig. 4a). Dogs who lived in households with a higher stability score were reported to be healthier than those that did not (β_stability_ = 0.044, *P* = 1.07 × 10^−45^; Fig. 4a). Dogs that lived in households with more children were reported to be less healthy than those with fewer children (β_children_ = −0.041, *P* = 2.72 × 10^−3^; Fig. 4a). Finally, older owners reported their dogs were in better health compared to younger owners (β_owner_age_ = 0.23, *P* = 5.60 × 10^−41^; Fig. 4a).

#### Disease diagnoses.

We found similar associations between environmental factors and health when using an arguably more objective measure of health: the total number of disease diagnoses per dog over their lifetime (the total count of all reported diagnosed diseases; see methods). All five factors were significantly associated with disease diagnoses, controlling for age and weight. Specifically, income was positively associated with the number of reported diseases, while stability, time with other animals, owner age and time with children were negatively associated with the number of diseases reported. Dogs in more stable households or with older owners were reported to have fewer diseases (β_stability_ = −0.03, *P* ≤ 2 × 10^−16^; β_owner_age_ = −0.16, *P* ≤ 2 × 10^−16^). For the two measures of social integration and connectedness, we found that dogs with more nonhuman companions as well as those with more children in the house had significantly fewer reported diseases (β_social-animals_ = −0.03, *P* = 9.09 × 10^−6^, β_social-children_ = −0.09, *P* ≤ 2 × 10^−16^) (Fig. 4b). Paradoxically, we observed a positive association between income and reported disease instances (β_income_ = 0.04, *P* = 2.27 × 10^−7^), which stood in contrast to the negative association between higher income and better owner-reported health.

#### Mobility.

Given that mobility declines with age and can be used as a marker for exposures that might alter the rate of aging, we investigated associations between the environmental factors and composite mobility score that captured measures of physical activity duration, intensity and frequency [55–57]. In this metric, a higher score reflected higher levels of activity and mobility (see Methods for details on the calculation of the composite mobility score). As expected, older dogs were less mobile (β_age_ = −0.12, *P* ≤ 2 × 10^−16^; SI Table 3), and, when controlling for age, we found that dogs with higher mobility were in better health compared to less mobile dogs of the same age (β = 0.35, *P* ≤ 2 × 10^−16^; SI Table 3). This suggests that, similar to humans, a more active lifestyle is associated with better health in dogs [58, 59]. We next found that the dog’s social environment—specifically stability, time spent with children and owner age—were significantly associated with dog mobility. Mobility was lower in dogs that lived in households with more children and higher for dogs that lived in households with older owners (β_children_ = −0.04, *P* ≤ 2 × 10^−16^, β_owner age_ = 0.03, *P* = 1.15 × 10^−14^, respectively), while it was higher in dogs in households that were more stable (β_stability_ = 4.55 × 10^-3^, *P* = 4.52 × 10^−12^; Fig. 4c, SI Table 3).

### Effects of environmental determinants of health can vary across the lifespan

**Figure 5.**
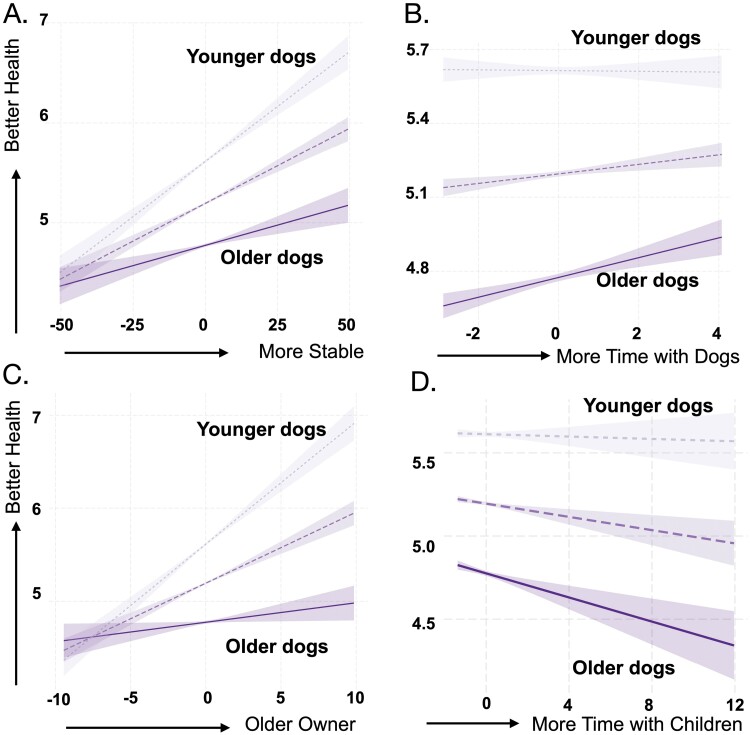
Age-dependent associations between environmental factors and health. (a) Household stability was more strongly associated with health for younger dogs compared to older dogs. (b) The presence of more social companions was more strongly associated with health for older dogs compared to younger dogs. (c) The factor of owner age was more strongly associated with health for younger dogs compared to older dogs. (d) More time spent with children was more strongly associated with health for older dogs compared to younger dogs. All graphs show predicted trends using a linear model framework with 95% confidence intervals for a hypothetical population of dogs aged 4 (−1 SD, lightest purple), 7.9 (mean age, intermediate purple) and 11.8 (+1 SD, darkest purple) years old

To investigate whether associations between these factors and health, disease and mobility vary across the lifespan, we extended each regression model by including an interaction term between each environmental factor and age (Supplementary Fig. 4). We found five significant interactions between dog age and our environmental factors on owner-reported dog health (SI Table 3). The positive association between household stability and dog health was strongest in the younger dogs, and weakest in older dogs (Fig. 5a, β = −0.03, *P* = 5.33 × 10^−17^). Time spent with other animals was more strongly associated with health when the dog was older rather than younger (Fig. 5b, β = 0.03, *P* = 4.18 × 10^−2^). We also found that owner age was more impactful for younger dogs compared to older dogs (Fig. 5c, β = −0.18, *P* = 5.91 × 10^−26^). Lastly, for older dogs, increased time spent with children was more impactful to overall general health than for younger dogs (Supplementary Fig. 6, β = −0.04, *P* = 1.01 × 10^−2^). We saw no significant interactions between environmental factors and age on disease instances.

We did find a significant interaction between dog age and four environmental factors on mobility. First, a significant interaction between neighborhood stability and dog age on mobility revealed that neighborhood stability is most strongly associated with the mobility of younger dogs compared to older dogs (β = −0.002, *P* = 6.0 × 10^−4^; Supplementary Fig. 5a; SI Table 3). Second, the effect of spending time with other animals on mobility significantly interacted with dog age, where more time was considerably more important for the mobility of older dogs (β = −0.01, *P* = 8.2 × 10^−4^, Supplementary Fig. 5b: SI Table 3). Third, the effect of spending time with children on mobility significantly interacted with dog age, where more time with children was more important for the mobility of older dogs (β = −0.01, *P* = 0.01, Supplementary Fig. 5c; SI Table 3). Lastly, having an older owner had a stronger effect on a younger dog’s mobility (β = −0.01, *P* = 7.9 × 10^−5^, Supplementary Fig. 5d).

### Pathways linking social-environmental variables and owner-reported dog health

Environmental factors could have both direct and indirect effects on health. To examine these putative paths, we took an orthogonal modeling approach, structural equation modeling (SEM), which can capture more complex relationships among the environmental variables and health. This approach can have more flexibility, the capacity to represent indirect relationships, and supports the integration of results into broader theory [51]. To do so, we first organized our variables into latent factors that represent concepts known to fundamentally influence mammalian health and that were chosen to maximize the interpretability of the latent factors [60]. We chose the variables (i.e. survey items) that were identified as most informative in our exploratory factor analysis described above. The latent factor of health was informed by the same variables that we used as outcome variables in our linear models: health, disease instances and mobility.

Both showed good model fit according to established parameters [52] (M1: CFI = 0.84, RMSEA = 0.06, SRMR = 0.05, TLI = 0.80; M2: CFI = 0.84, RMSEA= 0.06, SRMR = 0.05, TLI = 0.80). Here we will present results for M2, which includes a pathway from the financial factor to the environmental factor, which is a better representation of the system given the established literature [61, 62]. In humans, we know that the quality of the environment is influenced by the finances of the household thus providing support for evaluating M2 as the more generalizable model [63].

**Figure 6.**
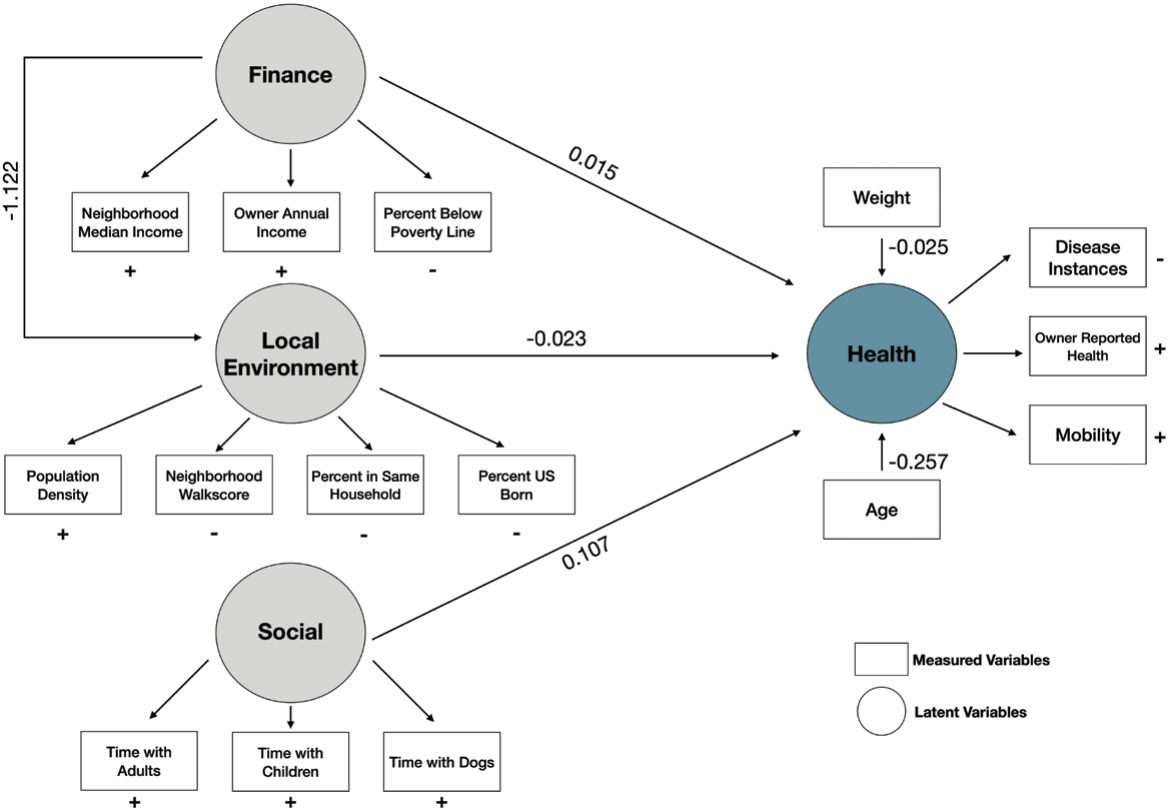
Structural equation modeling captures a complex network of environmental effects on health. Structural equation model of how the social environment can influence general health. Path coefficients explain the effect sizes and directionality of each major path. Latent factors (circles) are informed by measured variables (squares) that were chosen via factor analysis, and the direction of the effect of each measured variable on the latent variables is indicated next to or below the variable (+: positive association; −: negative association)

As with our linear models, we found evidence for all three latent variables associated with the latent factor capturing health. However, the relative importance of the latent factors varied: the effect of the social factor was ~5x larger than the effect of finance and the local environment on our health outcome (β_Social_ = 0.107, β_Finance_ = 0.015, β_Environment_ = −0.023; Fig. 6). In line with our linear models, we found that increased income and more social interaction with humans and other dogs both predicted better health. In contrast, a more urban environment with less stability was associated with worse health, pointing to the complex relationship between overall health and the environment. These data capture a previously hypothesized indirect pathway from finance through the local environment that our linear models failed to identify, suggesting that the local environment might be an important mediator between finance and health.

## DISCUSSION

Using the largest observational dataset of companion dog environment and health, we identified measures of the social and lived environment that were linked to age-associated changes in health, mobility and disease. Unsurprisingly, age was the strongest predictor of health deficits. However, not all dogs exhibited the same age-related changes in health, disease and mobility, and some of this inter-individual variation was linked to the environment. Health, mobility and disease measures were also associated with variations in household social environmental determinants. We also identified key changes in the environment that were associated with the health and mobility of dogs in an age-dependent manner within this cross-sectional dataset.

Similar to humans, we found that measures of income and social connectedness (i.e. presence of more potential social companions) predicted better health in dogs, although dogs from wealthier owners had more diagnosed diseases [64]. This counterintuitive association does not necessarily mean that dogs of wealthier owners had more diseases, especially given the fact that dogs from higher SES households were reported as healthier by their owners. Rather, this finding points to the role that finance plays in the opportunities for disease diagnosis, while also generating new questions on the directionality of the relationship between income and health as well as what we ultimately consider ‘health’ and how we diagnose diseases [60]. Dogs who live in households with wealthier owners might seek veterinary care more frequently and have the funds to pay for additional tests, resulting in more disease diagnoses even though the diseases are unlikely to be more prevalent in higher SES households. Owners may perceive their dogs to be in good health because they have more information and are more confident that any disease would have been detected and treated or managed. Dogs who lived in households with other pets had higher reported health scores and fewer disease diagnoses than dogs who had fewer household companions, suggesting that this social enrichment confers health benefits, which outweighed the effects of finances five-fold (Fig. 6). These other companions are often other dogs—of homes with other animals present, 70% of them had another dog—but could be other pets (e.g. cats, SI Table 7). This suggests that similar to humans, increased social enrichment in companion dogs is associated with health benefits, and also provides an example of an easily modifiable environmental intervention to improve dog welfare. These health benefits may also impact the owners. Indeed, a recent study found that interspecies companionship lowered frailty among older humans and may point to an effective intervention to improve the health of aging humans [65]. Dogs who lived in households with more children had fewer diseases, but tended to be in poorer health. This observation could be due to modified resource allocation (e.g. time and money) in households with more children. Mobility was also associated with environmental factors and showed unique age dependencies. This recapitulates what we see in humans, where individuals who live in lower-income neighborhoods and experience adverse community-level factors, such as decreased access to green space, unequal resource distribution and access (e.g. healthcare or food), have poorer health outcomes [66, 67].

We found that owner age was significantly associated with our health-related outcomes, but why this pattern occurs is unknown. One possible mechanism is the link between owner age and dog activity levels, where a recent analysis in the DAP found that older owners had more active dogs, which is linked to better health. Further answers to this question could elucidate the complex relationships between owner age and our other factors and their associations with health [68]. This suggests that as the DAP continues, investigators will need to pay particular attention to owner age in our dataset, as it has a potential association with physical activity, cognitive function and now perceived health.

An important outstanding question is identifying the path through which the social environment impacts health. In humans, it has been hypothesized (and there is some support for the fact) that exposure to psychosocial stressors from unpredictable environments (e.g. work-related stress, low SES, unpredictable environments and low social integration) lastingly alter the stress-response pathway and, consequently, immune function and health [69]. Our findings fit within this framework and show that the negative health effects of unpredictable social environments can extend to companion dogs. However, more work is needed to identify whether or not the physiological pathways (e.g. stress pathway) are shared among these two cohabitating species.

We also unexpectedly found that younger dogs with older owners experienced better health outcomes compared to older dogs who had older owners. These data suggest the importance of understanding how age impacts lifestyle, which could have knock-on effects for companions and companion animals. Specifically, the human social environment might contribute to dog health indirectly through variables like owner age that affect how often a dog might engage in social enrichment activities due to their owner’s activity budget and lifestyle. We also acknowledge that the distribution of dog age is highly non-random with respect to owner age, which may explain some of this association. We found that younger dogs who lived in less stable households were in worse health than older dogs who lived in stable homes, suggesting that, similar to humans, environmental adversity plays a particularly important role in early life [70–72]. Older dogs who lived in higher-income households were found to be in better health compared to older dogs in lower-income households, while the health of younger dogs was less impacted by income variation, suggesting that dogs may be more resilient to variation in this form of adversity earlier in life [73, 74]. Younger dogs’ mobility experienced a larger age-associated increase when living in more stable neighborhoods compared to older dogs, suggesting that the benefits of living in a more stable household, while important across the lifespan, are more impactful in earlier life. Older dogs who lived in less stable neighborhoods were much less mobile than older dogs in more stable neighborhoods, suggesting that, similar to older people in lower SES neighborhoods, dogs experience a reduction in activity that could ultimately be linked with poorer health outcomes [75, 76].

Our environmental and health measures are not independent of one another, suggesting a more complex network among environmental factors and their association with health outcomes. We used SEM to capture direct and indirect effects of each variable in one model. The results of our SEM supported the independent models of each health variable, but allowed us to propose a holistic model for the environmental impacts on companion dog health and aging that is generalizable to similar work done on humans. The model points to direct effects of finance and the local environment on dog health, in addition to an indirect contribution of finance on health through its effect on the local environment. This indirect path is supported in the human literature, where we see that SES dictates where a person will live and thus their environment as well as access to healthcare and both of these contribute to both positive and negative health outcomes [68, 77]. This indicates the latent structures affecting health outcomes in dogs and humans are similar, and may be a product of aspects of modern society as well as the deeper evolutionary history of dogs being selectively bred alongside humans for thousands of years.

While the strengths of this dataset include the large sample size (*n* = 21,410) of the study cohort and the breadth of the HLES survey data, there are some limitations. First, our dataset comprises both mixed-breed and purebred dogs, and genetic background and dog breed are both known to be associated with dog health and disease [20, 53]. However, our results remained largely consistent when we examined pure and mixed breed dogs independently, as well as when we controlled for individual breeds based on owner reports (SI Table 4). The Dog Aging Project is currently sequencing the genomes of 10,000 dogs in the project [16], which will allow us to more accurately control for the genetic background as well as identify putative gene-by-environment interactions that impact health, disease, or mobility.

Second, all data in the HLES are owner-reported and can thus be affected by subjective error, bias and/or misinterpretation of survey questions. Further investigation of biases in owner-reported responses will be needed to verify our findings in the future. The strong association between dog age and all three of our outcome measures—health, disease incidence and mobility—suggests that the survey is an instrument that, on average, accurately captures the variables we cannot objectively measure. All measures could be improved by an owner-independent measure or veterinarian-informed measure of frailty and health. In future research, we will draw on electronic veterinary medical records, molecular and immunological measures and at-home physical tests to generate more accurate measures of health and frailty in the companion dog.

Third, there is a limitation due to the average household income being well above the U.S. median income (U.S. median: $67,521 in 2020, DAP median: $100,000–$119,999 [78]) and biases our knowledge away from lower-earning households. This underscores the need to recruit a diverse set of human participants to better account for the variation of environmental exposures a dog is subject to [16].

Fourth, given the observational nature of the data, we were not able to determine causality or directionality among variables; we instead drew a causal structure in the SEM based on a critical evaluation of the literature (SI Table 6). For instance, owners who have dogs in poorer health might limit exposures to demanding activities or social enrichment compared to owners who have dogs who are in excellent health, which would also lead to an association between health and social companionship and activity.

Given the above limitations, the results of this analysis should not be used to influence medical decisions, behavioral interventions, or policy changes related to the care of companion dogs, but rather encourage the continued investigation by veterinary professionals and research scientists on best practices for increasing companion welfare. Some of these relationships can be evaluated in the future with randomized, controlled experiments.

Overall, our study provides further evidence for the strong link between the social environment and health outcomes that reflects what is known for humans. From this knowledge, we propose the companion dog to be a novel model for human aging. This highlights the need for more attention to the role of the social environment on health and disease and continued investigation of how each environmental factor can contribute to more years of healthy living (i.e. ‘healthspan’) in both companion dogs and humans. A holistic understanding of environmental effects on both humans and dogs may offer insight into what factors are important for health—independent of confounding factors that complicate human studies (e.g. smoking). Results highlight the need to study how differences in resource allocation among households contribute to the health and well-being of all members of the household. A future use of longitudinal data will allow a stronger inferential capacity to identify putative causal factors that affect trajectories of health and aging in both dogs and humans, driving new insights into what factors promote healthy aging and potential lifestyle modifications that can increase time spent free of disease.

Dog Aging Project Consortium Authors (as of March 2022)

Joshua M. Akey^1^, Brooke Benton^2^, Elhanan Borenstein^3^, Marta G. Castelhano^4^, Amanda E. Coleman^5^, Kate E. Creevy^6^, Kyle Crowder^7^, Matthew D. Dunbar^8^, Virginia R. Fajt^9^, Annette L. Fitzpatrick^10^, Unity Jeffery^11^, Erica C Jonlin^12^, Matt Kaeberlein^13^, Elinor K. Karlsson^14^, Kathleen F. Kerr^15^, Jonathan M. Levine^16^, Jing Ma^17^, Robyn L McClelland^18^, Daniel E.L. Promislow^19^, Audrey Ruple^20^, Stephen M. Schwartz^21^, Sandi Shrager^22^, Noah Snyder-Mackler^23^, Katherine Tolbert^24^, Silvan R. Urfer^25^, Benjamin S. Wilfond^26^

^1^ Lewis-Sigler Institute for Integrative Genomics, Princeton University, Princeton, NJ, USA

^2^ Department of Laboratory Medicine and Pathology, University of Washington School of Medicine, Seattle, WA, USA

^3^ Department of Clinical Microbiology and Immunology, Sackler Faculty of Medicine, Tel Aviv University, Tel Aviv, Israel

^4^ Cornell Veterinary Biobank, College of Veterinary Medicine, Cornell University, Ithaca, NY, USA

^5^ Department of Small Animal Medicine and Surgery, College of Veterinary Medicine, University of Georgia, Athens, GA, USA

^6^ Department of Small Animal Clinical Sciences, Texas A&M University College of Veterinary Medicine & Biomedical Sciences, College Station, TX, USA

^7^ Department of Sociology, University of Washington, Seattle, WA, USA

^8^ Center for Studies in Demography and Ecology, University of Washington, Seattle, WA, USA

^9^ Department of Veterinary Physiology and Pharmacology, Texas A&M University College of Veterinary Medicine & Biomedical Sciences, College Station, TX, USA

^10^ Department of Family Medicine, University of Washington, Seattle, WA, USA

^11^ Department of Veterinary Pathobiology, Texas A&M University College of Veterinary Medicine & Biomedical Sciences, College Station, TX, USA

^12^ Department of Laboratory Medicine and Pathology, University of Washington School of Medicine, Seattle, WA, USA

^13^ Department of Laboratory Medicine and Pathology, University of Washington School of Medicine, Seattle, WA, USA

^14^ Bioinformatics and Integrative Biology, University of Massachusetts Chan Medical School, Worcester, MA, USA

^15^ Department of Biostatistics, University of Washington, Seattle, WA, USA

^16^ Department of Small Animal Clinical Sciences, Texas A&M University College of Veterinary Medicine & Biomedical Sciences, College Station, TX, USA

^17^ Division of Public Health Sciences, Fred Hutchinson Cancer Research Center, Seattle, WA, USA

^18^ Department of Biostatistics, University of Washington, Seattle, WA, USA

^19^ Department of Laboratory Medicine and Pathology, University of Washington School of Medicine, Seattle, WA, USA

^20^ Department of Population Health Sciences, Virginia-Maryland College of Veterinary Medicine, Virginia Tech, Blacksburg, VA, USA

^21^ Epidemiology Program, Fred Hutchinson Cancer Research Center, Seattle, WA, USA

^22^ Collaborative Health Studies Coordinating Center, Department of Biostatistics, University of Washington, Seattle, WA, USA

^23^ School of Life Sciences, Arizona State University, Tempe, AZ, USA

^24^ Department of Small Animal Clinical Sciences, Texas A&M University College of Veterinary Medicine & Biomedical Sciences, College Station, TX, USA

^25^ Department of Laboratory Medicine and Pathology, University of Washington School of Medicine, Seattle, WA, USA

^26^ Treuman Katz Center for Pediatric Bioethics, Seattle Children’s Research Institute, Seattle, WA, USA

## Supplementary Data

Supplementary data is available at *EMPH* online.

**Supplementary Figure 1.** Distribution of owner ages (a), distribution of owner education level (b), distribution of owner income range (c), distribution of neighborhood median income (d) and distribution of neighborhood unemployment rate (e)

**Supplementary Figure 2.** M1

**Supplementary Figure 3.** M2

**Supplementary Figure 4.** Ordinal, linear, and generalized linear models for each outcome and analyses

**Supplementary Figure 5.** (a) The association between dog mobility and household stability varied with dog age. (b) The association between dog mobility and time spent with dogs varied with dog age. (c) The association between dog mobility and time spent with children varied with dog age. (d) The association between dog mobility and owner age varied with dog age

**Supplementary Figure 6.** The association between overall dog health and time spent with children varied with dog age

**SI Table 1.** Dog and owner demographics

**SI Table 2.** Variables

**SI Table 3.** Main effects models

**SI Table 4.** Models for pure/mixed

**SI Table 5.** Disease metric

**SI Table 6.** SEM variables

**SI Table 7.** Other animals

**SI Table 8.** Ordinal model vs linear model

**SI Table 9.** Ordinal model with dogs ≤ 20yrs

**SI Table 10.** SEM model output

eoad011_suppl_Supplementary_Figure_S1

eoad011_suppl_Supplementary_Figure_S2

eoad011_suppl_Supplementary_Figure_S3

eoad011_suppl_Supplementary_Figure_S4

eoad011_suppl_Supplementary_Figure_S5

eoad011_suppl_Supplementary_Figure_S6

eoad011_suppl_Supplementary_Tables

## Data Availability

All code to reproduce the analyses can be found here: https://github.com/smacklab/dap_social_determinants_2023. The raw data are housed on the Terra platform at the Broad Institute of MIT and Harvard and access to it can be requested here: https://dogagingproject.org/open_data_access/

## References

[1] Snyder-Mackler N , Burger JR, Gaydosh L et al. Social determinants of health and survival in humans and other animals. Science 2020;368. DOI: 10.1126/science.aax9553. PMC7398600 32439765

[2] Weyers S , Dragano N, Möbus S et al. Low socio-economic position is associated with poor social networks and social support: results from the Heinz Nixdorf Recall Study. Int J Equity Health 2008;7:13. DOI: 10.1186/1475-9276-7-13. 18457583 PMC2424055

[3] Holt-Lunstad J , Smith TB, Layton JB. Social relationships and mortality risk: a meta-analytic review. PLoS Med 2010;7:e1000316. DOI: 10.1371/journal.pmed.1000316. 20668659 PMC2910600

[4] Algren MH , Ekholm O, Nielsen L et al. Social isolation, loneliness, socioeconomic status, and health-risk behaviour in deprived neighbourhoods in Denmark: a cross-sectional study. SSM Popul Health 0546;2020:10. DOI: 10.1016/j.ssmph.2020.100546. PMC6997896 32042889

[5] Hawkley LC , Cole SW, Capitanio JP et al. Effects of social isolation on glucocorticoid regulation in social mammals. Horm Behav 2012;62:314–23. DOI: 10.1016/j.yhbeh.2012.05.011. 22663934 PMC3449017

[6] Sullens DG , Gilley K, Jensen K et al. Social isolation induces hyperactivity and exploration in aged female mice. PLoS One 2021;16:e0245355. DOI: 10.1371/journal.pone.0245355. 33534853 PMC7857591

[7] Razzoli M , Nyuyki-Dufe K, Gurney A et al. Social stress shortens lifespan in mice. Aging Cell 2018;17:e12778. DOI: 10.1111/acel.12778. 29806171 PMC6052478

[8] Scott SB , Sliwinski MJ, Blanchard-Fields F. Age differences in emotional responses to daily stress: the role of timing, severity, and global perceived stress. Psychol Aging 2013;28:1076–87. DOI: 10.1037/a0034000. 24364410 PMC3874135

[9] Sarkar C , Webster C. Urban environments and human health: current trends and future directions. Curr Opin Environ Sustain 2017;25:33–44. DOI: 10.1016/j.cosust.2017.06.001.

[10] Vlahov D , Freudenberg N, Proietti F et al. Urban as a determinant of health. J Urban Health 2007;84:16–26. DOI: 10.1007/s11524-007-9169-3. PMC1891649 17356903

[11] Zaninotto ASA. Lower socioeconomic status and the acceleration of aging: an outcome-wide analysis. PNAS 2020;117:14911–7. DOI: 10.1073/pnas.1915741117. 32541023 PMC7334539

[12] Zeng Y , Gu D, Purser J et al. Associations of environmental factors with elderly health and mortality in China. Am J Public Health 2010;100:298–305. DOI: 10.2105/AJPH.2008.154971. 20019314 PMC2804639

[13] Hadley MB , Nalini M, Adhikari S et al. Spatial environmental factors predict cardiovascular and all-cause mortality: results of the SPACE study. PLoS One 2022;17:e0269650. DOI: 10.1371/journal.pone.0269650. 35749347 PMC9231727

[14] Ruple A , MacLean E, Snyder-Mackler N et al. Dog models of aging. Annu Rev Anim Biosci 2022;10:419–39. DOI: 10.1146/annurev-animal-051021-080937. 34699257 PMC8962603

[15] Hoffman JM , Creevy KE, Franks A et al. The companion dog as a model for human aging and mortality. Aging Cell 2018;17:e12737. DOI: 10.1111/acel.12737. 29457329 PMC5946068

[16] Creevy KE , Akey JM, Kaeberlein M et al.; Dog Aging Project Consortium. An open science study of ageing in companion dogs. Nature 2022;602:51–7. DOI: 10.1111/acel.12737. 35110758 PMC8940555

[17] Gilmore KM , Greer KA. Why is the dog an ideal model for aging research? Exp Gerontol 2015;71:14–20. DOI: 10.1016/j.exger.2015.08.008. 26325590

[18] Mazzatenta A , Carluccio A, Robbe D et al. The companion dog as a unique translational model for aging. Semin Cell Dev Biol 2017;70:141–53. DOI: 10.1016/j.semcdb.2017.08.024. 28803893

[19] Dias-Pereira P. Morbidity and mortality in elderly dogs—a model for human aging. BMC Vet Res 2022;18:1–8. DOI: 10.1186/s12917-022-03518-8. 36581919 PMC9798575

[20] Fleming JM , Creevy KE, Promislow DEL. Mortality in north American dogs from 1984 to 2004: an investigation into age-, size-, and breed-related causes of death. J Vet Intern Med 2011;25:187–98. DOI: 10.1111/j.1939-1676.2011.0695.x. 21352376

[21] Vaysse A , Ratnakumar A, Derrien T et al.; LUPA Consortium. Identification of genomic regions associated with phenotypic variation between dog breeds using selection mapping. PLoS Genet 2011;7:e1002316. DOI: 10.1371/journal.pgen.1002316. 22022279 PMC3192833

[22] Vonholdt BM , Pollinger JP, Lohmueller KE et al. Genome-wide SNP and haplotype analyses reveal a rich history underlying dog domestication. Nature 2010;464:898–902. DOI: 10.1038/nature08837. 20237475 PMC3494089

[23] Shearin AL , Ostrander EA. Leading the way: canine models of genomics and disease. Dis Model Mech 2010;3:27–34. DOI: 10.1242/dmm.004358. 20075379 PMC4068608

[24] Karlsson EK , Lindblad-Toh K. Leader of the pack: gene mapping in dogs and other model organisms. Nat Rev Genet 2008;9:713–25. DOI: 10.1038/nrg2382. 18714291

[25] Whitson HE , Cohen HJ, Schmader KE et al. Physical resilience: not simply the opposite of frailty. J Am Geriatr Soc 2018;8:1459–61. DOI: 10.1111/jgs.15233. PMC6157007 29577234

[26] Whitson HE , Duan-Porter W, Schmader KE et al. Physical resilience in older adults: systematic review and development of an emerging construct. J Gerontol A Biol Sci Med Sci 2016;71:489–95. DOI: 10.1093/gerona/glv202. 26718984 PMC5014191

[27] Candela Andrade M , Slunsky P, Klass LG et al. Risk factors and long-term surgical outcome of patellar luxation and concomitant cranial cruciate ligament rupture in small breed dogs. Vet Med 2020;65:159–67. DOI: 10.17221/155/2019-VETMED. PMC11334444 39170807

[28] Allenspach K , Wieland B, Gröne A et al. Chronic enteropathies in dogs: evaluation of risk factors for negative outcome. J Vet Intern Med 2007;21:700–8. DOI: 10.1892/0891-6640(2007)21[700:ceideo]2.0.co;2. 17708389

[29] Gillman MW. Developmental origins of health and disease. N Engl J Med 2005;353:1848–50. DOI: 10.1056/NEJMe058187. 16251542 PMC1488726

[30] Vargas J , Junco M, Gomez C et al. Early life stress increases metabolic risk, HPA axis reactivity, and depressive-like behavior when combined with postweaning social isolation in rats. PLoS One 2016;11:e0162665. DOI: 10.1371/journal.pone.0162665. 27611197 PMC5017766

[31] Pesonen A-K , Räikkönen K, Heinonen K et al. Depressive symptoms in adults separated from their parents as children: a natural experiment during World War II. Am J Epidemiol 2007;166:1126–33. DOI: 10.1093/aje/kwm254. 17875582

[32] Heim C , Newport DJ, Mletzko T et al. The link between childhood trauma and depression: insights from HPA axis studies in humans. Psychoneuroendocrinology 2008;33:693–710. DOI: 10.1016/j.psyneuen.2008.03.008. 18602762

[33] Pienaar AE. The association between under-nutrition, school performance and perceptual motor functioning in first-grade South African learners: the North-West Child Health Integrated with Learning and Development study. Health SA 2019;24:1046. DOI: 10.4102/hsag.v24i0.1046. 31934401 PMC6917456

[34] Houpt KA , Zicker S. Dietary effects on canine and feline behavior. Vet Clin North Am Small Anim Pract 2003;33:405–16, vii. DOI: 10.1016/s0195-5616(02)00115-8. 12701518

[35] Bisset ES , Howlett SE. The biology of frailty in humans and animals: understanding frailty and promoting translation. Aging Med (Milton) 2019;2:27–34. DOI: 10.1002/agm2.12058. 31942510 PMC6880675

[36] Chen FL , Ullal TV, Graves JL et al. Evaluating instruments for assessing healthspan: a multi-center cross-sectional study on health-related quality of life (HRQL) and frailty in the companion dog. bioRxiv 2022:2022.07.21.500746. DOI: 10.1101/2022.07.21.500746. PMC10651603 36781597

[37] Rantakokko M , Mänty M, Rantanen T. Mobility decline in old age. Exerc Sport Sci Rev 2013;41:19–25. DOI: 10.1097/JES.0b013e3182556f1e. 23038241

[38] Morgan EM , Heseltine JC, Levine GJ et al. Evaluation of a low-technology system to obtain morphological and mobility trial measurements in dogs and investigation of potential predictors of canine mobility. Am J Vet Res 2019;80:670–9. DOI: 10.2460/ajvr.80.7.670. 31246119 PMC7311064

[39] Kaeberlein M , Creevy KE, Promislow DEL. The dog aging project: translational geroscience in companion animals. Mamm Genome 2016;27:279–88. DOI: 10.1007/s00335-016-9638-7. 27143112 PMC4936929

[40] Creevy KE , Akey JM, Kaeberlein M et al. Author correction: an open science study of ageing in companion dogs. Nature 2022;608:E33. DOI: 10.1038/s41586-022-05179-x. 35941194

[41] Harris PA , Taylor R, Minor BL et al.; REDCap Consortium. The REDCap consortium: building an international community of software platform partners. J Biomed Inform 2019;95:103208. DOI: 10.1016/j.jbi.2019.103208. 31078660 PMC7254481

[42] Harris PA , Taylor R, Thielke R et al. Research electronic data capture (REDCap)—a metadata-driven methodology and workflow process for providing translational research informatics support. J Biomed Inform 2009;42:377–81. DOI: 10.1016/j.jbi.2008.08.010. 18929686 PMC2700030

[43] Creevy KE , Grady J, Little SE et al. 2019 AAHA Canine Life Stage guidelines. J Am Anim Hosp Assoc 2019;55:267–90. DOI: 10.5326/JAAHA-MS-6999. 31622127

[44] Bray EE , Zheng Z, Tolbert MK et al.; Dog Aging Project Consortium. Once-daily feeding is associated with better health in companion dogs: results from the Dog Aging Project. Geroscience 2022;44:1779–90. DOI: 10.1007/s11357-022-00575-7. 35484470 PMC9213604

[45] Fabrigar LR , Wegener DT, MacCallum RC et al. Evaluating the use of exploratory factor analysis in psychological research. Psychol Methods 1999:272–99. DOI: 10.1037/1082-989x.4.3.272.

[46] Website. Available: Revelle W (2021). psych: Procedures for Psychological, Psychometric, and Personality Research. Northwestern University, Evanston, Illinois. R package version 2.1.9, https://CRAN.R-project.org/package=psych.

[47] Kraus C , Pavard S, Promislow DEL. The size-life span trade-off decomposed: why large dogs die young. Am Nat 2013;181:492–505. DOI: 10.1086/669665. 23535614

[48] Rosseel Y. lavaan: AnRPackage for structural equation modeling. J Stat Softw 2012. DOI: 10.18637/jss.v048.i02.

[49] Marmot M , Wilkinson RG. Social determinants of health in older age. Social Determinants of Health. Oxford: Oxford University Press, 2005, 267–96. DOI: 10.1093/acprof:oso/9780198565895.003.13.

[50] Jian Wang LG. Effects of socioeconomic status on physical and psychological health: lifestyle as a mediator. Int J Environ Res Public Health 2019;16:281–281. DOI: 10.3390/ijerph16020281. 30669511 PMC6352250

[51] Grace JB , Schoolmaster DR, Guntenspergen GR et al. Guidelines for a graph-theoretic implementation of structural equation modeling. Ecosphere 2012;3:1-44. DOI: 10.1890/es12-00048.1.

[52] Hu L , Bentler PM. Cutoff criteria for fit indexes in covariance structure analysis: Conventional criteria versus new alternatives. Struct Equ Modeling 2009;6:1–55. DOI: 10.1080/10705519909540118.

[53] Yordy J , Kraus C, Hayward JJ et al. Body size, inbreeding, and lifespan in domestic dogs. Conserv Genet 2020;21:137–148. DOI: 10.1007/s10592-019-01240-x. 32607099 PMC7326369

[54] German AJ. The growing problem of obesity in dogs and cats. J Nutr 2006;136:1940S–6S. DOI: 10.1093/jn/136.7.1940S. 16772464

[55] Ferrucci L , Cooper R, Shardell M et al. Age-related change in mobility: perspectives from life course epidemiology and geroscience. J Gerontol A Biol Sci Med Sci 2016;71:1184–94. DOI: 10.1093/gerona/glw043. 26975983 PMC4978365

[56] Guralnik JM , Ferrucci L, Simonsick EM et al. Lower-extremity function in persons over the age of 70 years as a predictor of subsequent disability. N Engl J Med 1995;332:556–62. DOI: 10.1056/NEJM199503023320902. 7838189 PMC9828188

[57] Studenski S , Perera S, Patel K et al. Gait speed and survival in older adults. JAMA 2011;305:50–8. DOI: 10.1001/jama.2010.1923. 21205966 PMC3080184

[58] Powell KE , Paluch AE, Blair SN. Physical activity for health: what kind? how much? how intense? on top of what? Annu Rev Public Health 2011;32:349–65. DOI: 10.1146/annurev-publhealth-031210-101151. 21128761

[59] Lee IM , Shiroma EJ, Lobelo F et al. Effect of physical inactivity on major non-communicable diseases worldwide: an analysis of burden of disease and life expectancy. Lancet 2012;380:219–29. DOI: 10.1016/S0140-6736(12)61031-9. 22818936 PMC3645500

[60] Daly M , Boyce C, Wood A. A social rank explanation of how money influences health. Health Psychol 2015;34:222–30. DOI: 10.1037/hea0000098. 25133843 PMC4507513

[61] Barrington WE. Neighborhood environment, stress, and obesogenic behaviors among adults. 2012. DOI: 10.1016/j.amepre.2014.08.025.

[62] Elliott M . The stress process in neighborhood context. Health Place 2000;6:287–99. DOI: 10.1016/s1353-8292(00)00010-1. 11027954

[63] Evans and GW, Kantrowitz E. Socioeconomic status and health: the potential role of environmental risk exposure. Ann Rev Public Health 2002;23:303-31.DOI: 10.1146/annurev.publhealth.23.112001.112349 11910065

[64] Simandan D. Rethinking the health consequences of social class and social mobility. Soc Sci Med 2018;200:258–61. DOI: 10.1016/j.socscimed.2017.11.037. 29301638

[65] Taniguchi Y , Seino S, Nishi M et al. Association of dog and cat ownership with incident frailty among community-dwelling elderly Japanese. Sci Rep 2019;9:1–7. DOI: 10.1038/s41598-019-54955-9. 31819092 PMC6901519

[66] Diez Roux AV , Mair CF. Neighborhoods and health. Ann N Y Acad Sci 2010;1186:125–45. DOI: 10.1111/j.1749-6632.2009.05333.x. 20201871

[67] Grafova IB , Freedman VA, Kumar R et al. Neighborhoods and obesity in later life. Am J Public Health 2008;98:2065–71. DOI: 10.2105/ajph.2007.127712. 18799770 PMC2636421

[68] Balia S , Jones AM. Mortality, lifestyle and socio-economic status. J Health Econ 2008;27:1–26. DOI: 10.1016/j.jhealeco.2007.03.001. 17448554

[69] Rohleder N. Stimulation of systemic low-grade inflammation by psychosocial stress. Psychosom Med 2014;76:181–9. DOI: 10.1097/PSY.0000000000000049. 24608036

[70] Nelson CA , Scott RD, Bhutta ZA et al. Adversity in childhood is linked to mental and physical health throughout life. BMJ 2020;371:m3048. DOI: 10.1136/bmj.m3048. 33115717 PMC7592151

[71] Suglia SF , Campo RA, Brown AGM et al. Social determinants of cardiovascular health: early life adversity as a contributor to disparities in cardiovascular diseases. J Pediatr 2020;219:267–73. DOI: 10.1016/j.jpeds.2019.12.063. 32111376 PMC7883398

[72] McCrory C , Dooley C, Layte R et al. The lasting legacy of childhood adversity for disease risk in later life. Health Psychol 2015;34:687–96. DOI: 10.1037/hea0000147. 25150540 10.1037/hea0000147

[73] Belsky J. The differential susceptibility hypothesis: sensitivity to the environment for better and for worse. JAMA Pediatr 2016;170:321–2. DOI: 10.1001/jamapediatrics.2015.4263. 26831915

[74] Esther Nederhof MVS. Mismatch or cumulative stress: Toward an integrated hypothesis of programming effects. Physiol Behav 2012;106:691–700. DOI: 10.1016/j.physbeh.2011.12.008. 22210393 10.1016/j.physbeh.2011.12.008

[75] Talaei M , Rabiei K, Talaei Z et al. Physical activity, sex, and socioeconomic status: a population based study. ARYA Atheroscler 2013;9:51–60. PMID: 23696760; PMCID: PMC3653259. 23696760 PMC3653259

[76] Powell LM , Slater S, Chaloupka FJ et al. Availability of physical activity-related facilities and neighborhood demographic and socioeconomic characteristics: a national study. Am J Public Health 2006;96:1676–80. PMID: 16873753; PMCID: PMC1551946 16873753 10.2105/AJPH.2005.065573 PMC1551946

[77] Contoyannis P , Jones AM. Socio-economic status, health and lifestyle. J Health Econ 2004;23:965–95. DOI: 10.1016/j.jhealeco.2004.02.001. 15353189

[78] US Census Bureau. Income and Poverty in the United States: 2020. 2021 [cited 16 Dec 2021]. Available: https://www.census.gov/library/publications/2021/demo/p60-273.html

