# Supplementary figures and images for: Social determinants of health and disease in companion dogs: a cohort study from the Dog Aging Project
Source: Evol Med Public Health. 2023 May 13;11(1):187–201. doi: 10.1093/emph/eoad011 (PMC10306367; doi:10.1093/emph/eoad011)

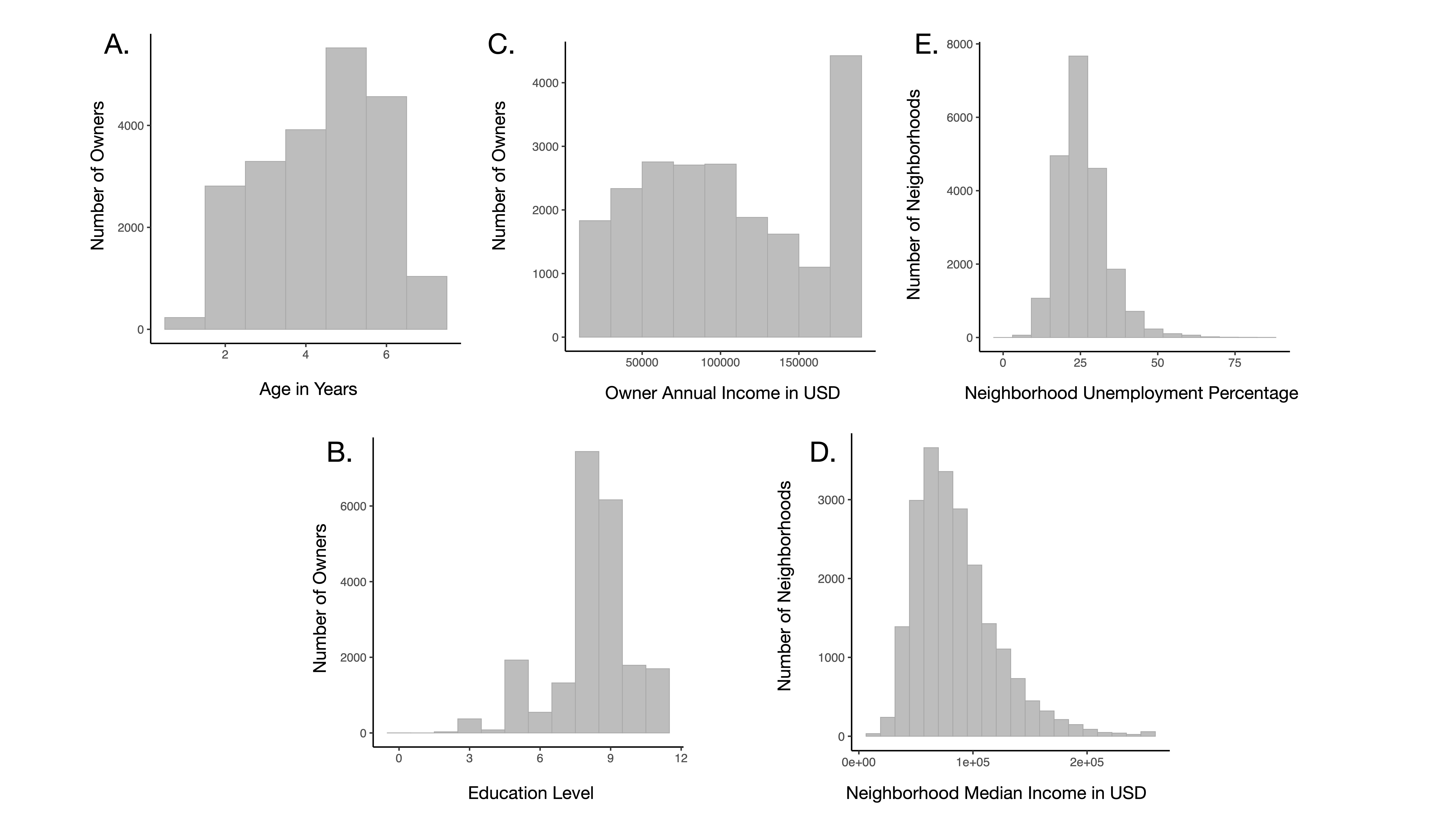

Supplement: eoad011_suppl_Supplementary_Figure_S1 [file eoad011_suppl_supplementary_figure_s1.jpeg]

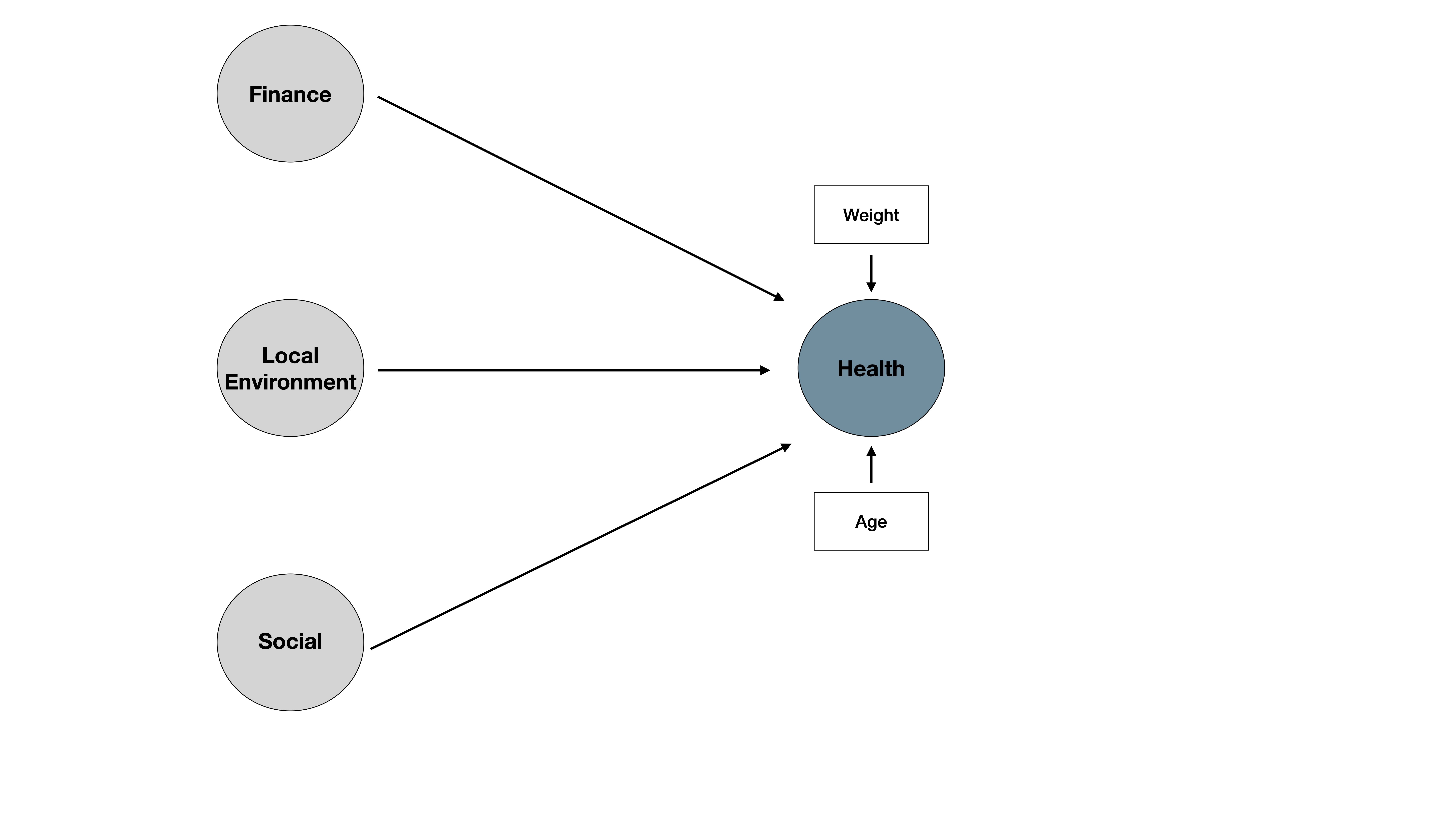

Supplement: eoad011_suppl_Supplementary_Figure_S2 [file eoad011_suppl_supplementary_figure_s2.jpeg]

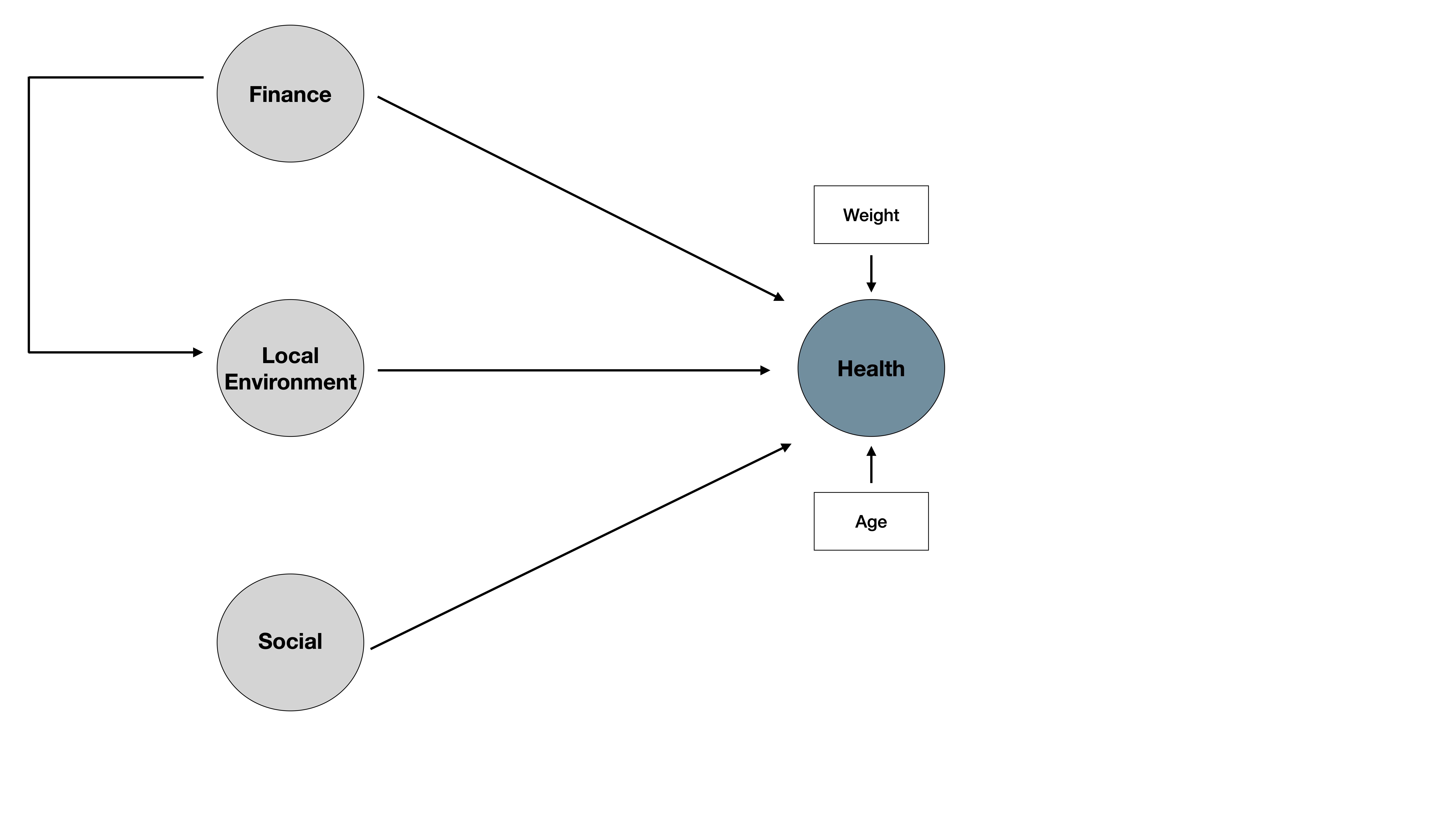

Supplement: eoad011_suppl_Supplementary_Figure_S3 [file eoad011_suppl_supplementary_figure_s3.jpeg]

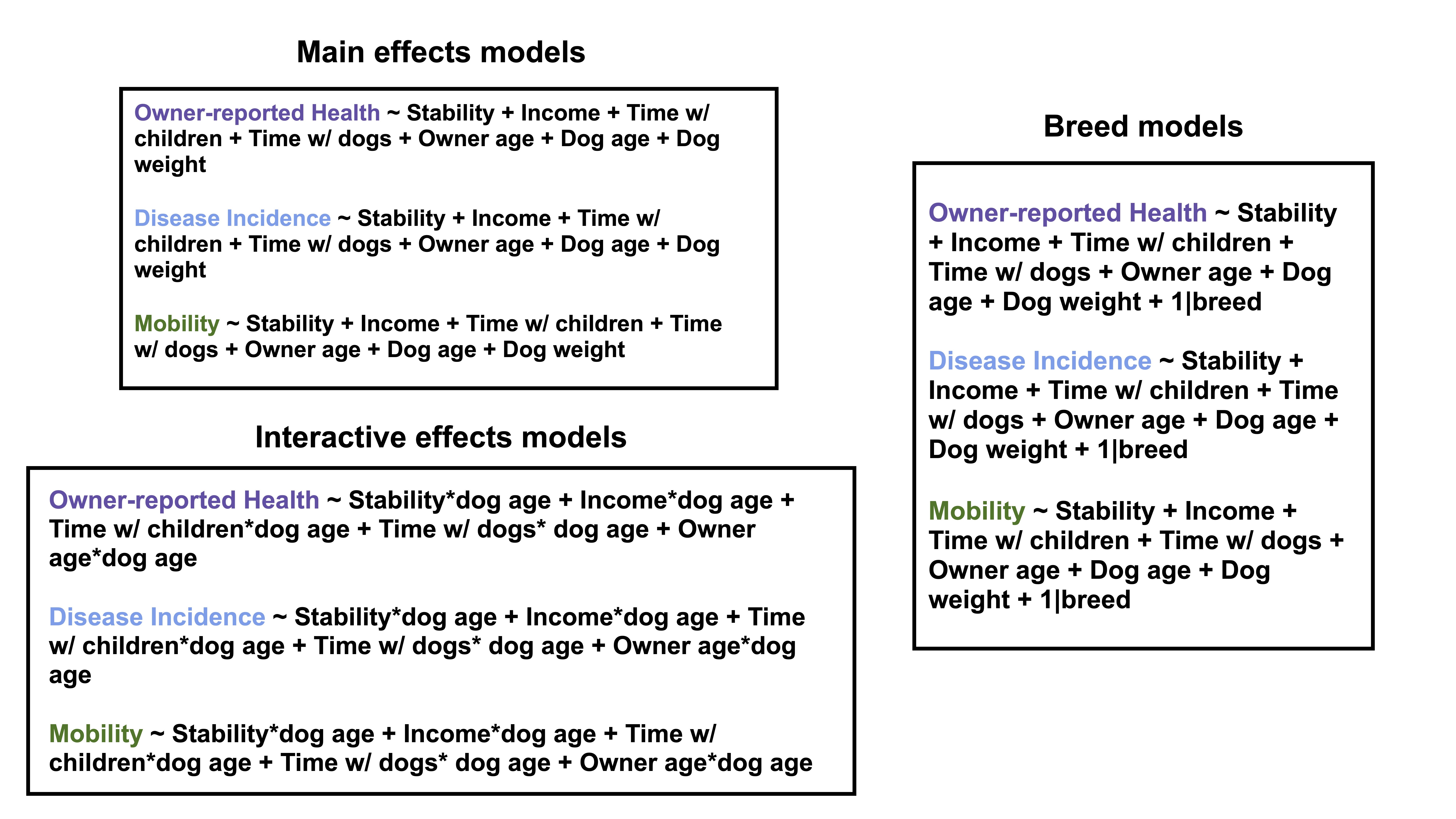

Supplement: eoad011_suppl_Supplementary_Figure_S4 [file eoad011_suppl_supplementary_figure_s4.jpeg]

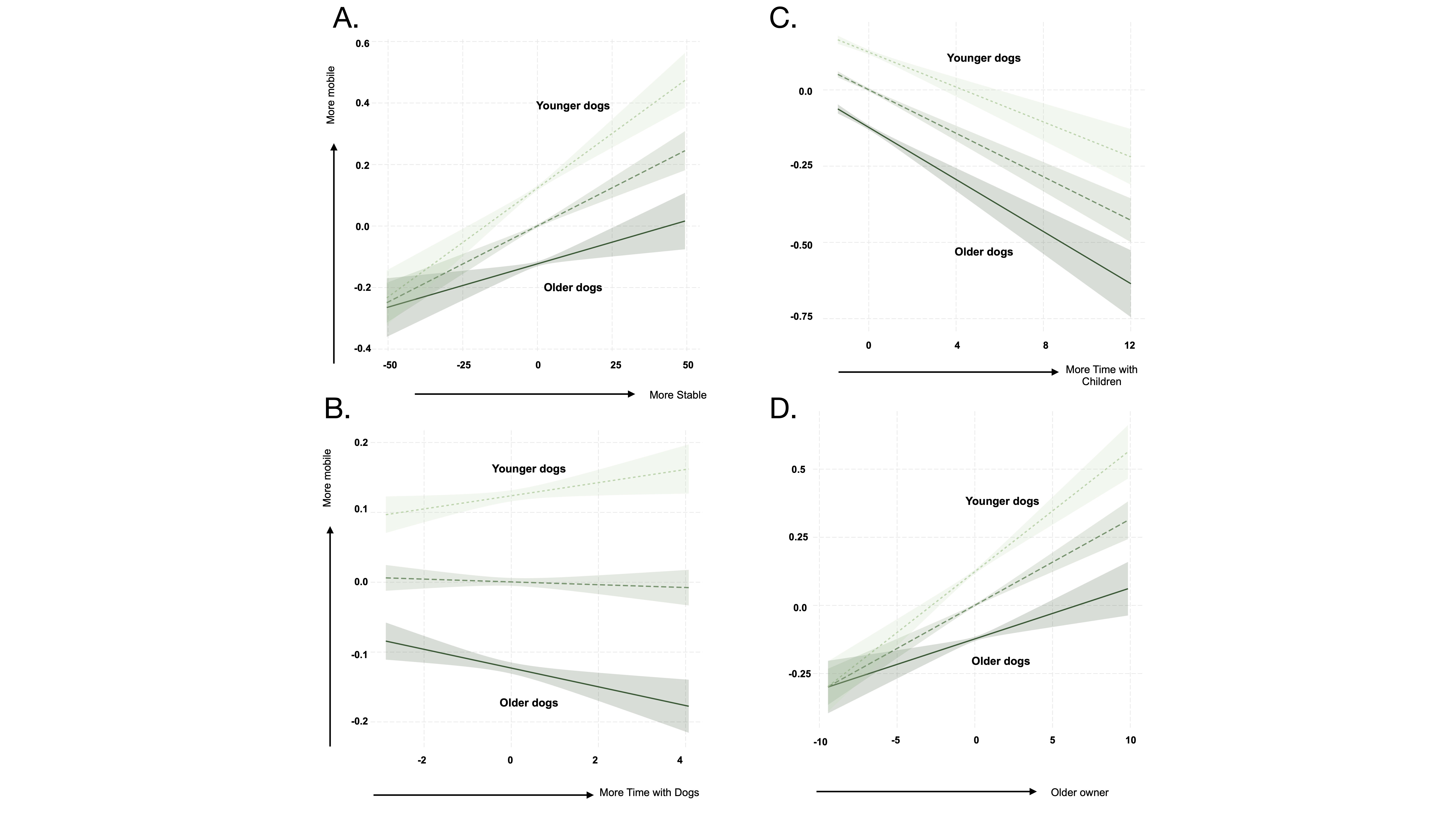

Supplement: eoad011_suppl_Supplementary_Figure_S5 [file eoad011_suppl_supplementary_figure_s5.jpeg]

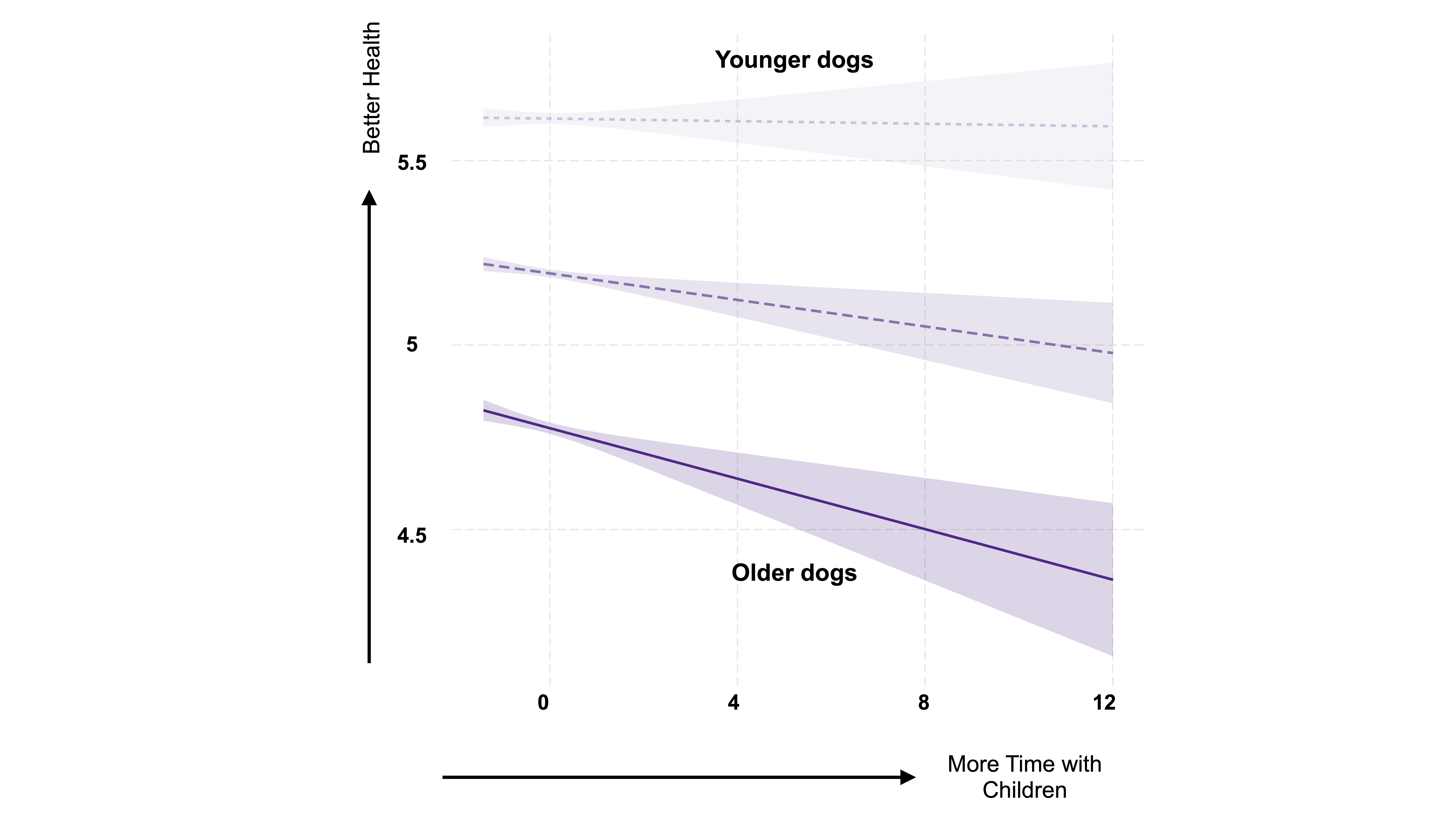

Supplement: eoad011_suppl_Supplementary_Figure_S6 [file eoad011_suppl_supplementary_figure_s6.jpeg]
